# Synergistic Effects and Mechanistic Insights into the Co-Hydropyrolysis of Chilean Oak and Polyethylene: Unlocking the Potential of Biomass–Plastic Valorisation

**DOI:** 10.3390/polym15122747

**Published:** 2023-06-20

**Authors:** Bastián Puentes, Fidel Vallejo, Serguei Alejandro-Martín

**Affiliations:** 1 Laboratory of Gas Chromatography and Analytical Pyrolysis, Universidad del Bío-Bío, Concepción 4030000, Chile; bastian.puentes1601@alumnos.ubiobio.cl (B.P.); fvallejo@ubiobio.cl (F.V.); 2 Wood Engineering Department, Faculty of Engineering, Universidad del Bío-Bío, Concepción 4030000, Chile

**Keywords:** biomass co-hydropyrolysis, thermogravimetric analysis, synergistic effect, synergy coefficient, Py-GC/MS, residues valorization

## Abstract

This study employed a hydrogen atmosphere in an analytical reactor to investigate the thermochemical transformation of Chilean Oak (ChO) and polyethylene. Thermogravimetric assays and compositional analyses of the evolved gaseous chemicals provided valuable insights regarding the synergistic effects during the co-hydropyrolysis of biomass and plastics. A systematic experimental design approach assessed the contributions of different variables, revealing the significant influence of the biomass/plastic ratio and hydrogen pressure. Analysis of the gas phase composition showed that co-hydropyrolysis with LDPE resulted in lower levels of alcohols, ketones, phenols, and oxygenated compounds. ChO exhibited an average oxygenated compound content of 70.13%, while LDPE and HDPE had 5.9% and 1.4%, respectively. Experimental assays under specific conditions reduced ketones and phenols to 2–3%. Including a hydrogen atmosphere during co-hydropyrolysis contributes to enhanced reaction kinetics and reduced formation of oxygenated compounds, indicating its beneficial role in improving reactions and diminishing the production of undesired by-products. Synergistic effects were observed, with reductions of up to 350% for HDPE and 200% for LDPE compared to the expected values, achieving higher synergistic coefficients with HDPE. The proposed reaction mechanism provides a comprehensive understanding of the simultaneous decomposition of biomass and polyethylene polymer chains, forming valuable bio-oil products and demonstrating the how the hydrogen atmosphere modulates and influences the reaction pathways and product distribution. For this reason, the co-hydropyrolysis of biomass–plastic blends is a technique with great potential to achieve lower levels of oxygenated compounds, which should be further explored in subsequent studies to address scalability and efficiency at pilot and industrial levels.

## 1. Introduction

Global energy demand has caused a sustained increase in fossil fuel consumption. Data from the World Bank updated to 2021 indicate that more than 159 peta-kWh of primary energy is consumed worldwide [1]. Consequently, about 34 billion tons of CO_2_ and other greenhouse gases contribute to climate change [2]. Projections indicate that global energy consumption will continue to grow at a rate of 2% annually until 2030, and this increase could be even higher due to anticipated lifestyle changes and population growth, with figures estimated to reach 8.6 billion people by 2030 [3,4]. Since 2010, the percentage contributed by fossil fuels worldwide has not decreased from 80%, which generates greenhouse gases. In addition, it produces other pollutants, such as nitrogen and sulfur oxides, which affect the environment and the long-term health of people. Despite this, notable milestones have been achieved in recent years in utilizing renewable energy. For instance, in the United States, in 2019, the consumption of renewable energies exceeded coal demand for the first time. This achievement signifies a significant shift towards cleaner and more sustainable energy sources [5,6].

Plastic waste poses a grave threat to marine ecosystems, with a projected tripling of spills in the next 20 years without intervention. Ocean plastic estimates range from 75 to 199 million tons, set to rise from 14 million tons (2016) to 37 million tons by 2040 [3]. Microplastics disrupt the carbon cycle, impacting primary production across ecosystems. Managing plastic waste incurs significant costs, projected to reach USD 100 billion in two decades [1]. Recycling rates lag despite a four-fold increase in global plastic production over 40 years. Shockingly, less than 10% of plastic waste is recycled, with most of it being transported far away for incineration or water disposal. Plastic packaging waste generates annual economic losses of USD 80–120 billion. Biodegradable plastics also pose challenges, persisting for years in the ocean. Urgent strategies are needed to address plastic waste and mitigate its environmental and economic impact [7]. 

The search for sustainable energy sources and the valorization of carbon-based waste have gained momentum in recent decades. Biomass, as a renewable and nearly carbon-neutral resource, offers excellent potential for replacing fossil fuels and producing alternative biofuels. It is an essential energy source with significant contributions to developed and developing economies [8]. Bioenergy involves the conversion of biomass into valuable energy through biological or thermochemical processes [9]. Thermochemical technologies, known for their high reaction efficiency and shorter processing times, are commonly preferred. Pyrolysis, a well-studied technology, is crucial in converting biomass into biofuels, particularly for various lignocellulosic substrates [10,11,12]. However, due to the high content of oxygenated compounds, bio-oils have an inadequate quality and low calorific value, which is a significant disadvantage [13]. The generation of oxygenated compounds is enhanced by lignocellulosic biomass’ low hydrogen content (3–7%). Two strategies have been suggested to improve the process. The first is the addition of plastics such as polypropylene (C_3_H_6_)_n_ and high-density (HDPE) and low-density polyethylene (C_2_H_4_)_n_ (LDPE), which have percentages of hydrogen close to 15%. The second is to increase the amount of hydrogen by injecting pure H_2_ into the reactor [14], a process known as co-hydropyrolysis. 

Recent studies have underlined plastic pyrolysis as a suitable route for effectively recovering energy from waste plastics. To provide hydrogen for pyrolysis reactions, plastics with high H and C contents and low oxygen content are preferred [15]. Due to their production volume, the most common thermoplastics are low-density polyethylene (LDPE), used in containers, bags, soft bottles, and laboratory materials, and high-density polyethylene (HDPE), which comes from pipes, toys, and bottles. HDPE is a cheap thermoplastic and is the fourth most produced plastic worldwide in volume after polypropylene (PP), LDPE, and polyvinyl chloride (PVC) [16]. It has a high strength–density ratio due to its higher density, which gives it stronger intermolecular forces than LDPE.

One of the main advantages of using thermoplastics that contain only C and H, such as polyethylene, is that it avoids the generation of dioxins [17]. Wang et al., 2021 used polyvinyl chloride (PVC) and studied the release of chlorine and dioxins during thermochemical conversion. They also explored the use of polystyrene (PS) and the production of toluene, ethylbenzene, and naphthalene through fast catalytic pyrolysis [18]. Polyethylene terephthalate (PET) has a similar amount of oxygen as most biomasses, with an equal H/Ceff value, but it is not biodegradable. Despite having the highest production within thermoplastics by volume and accounting for about 23% of plastic consumption worldwide, PP was found to have a high proportion of oxygenated compounds in a co-pyrolysis study with lignocellulosic biomass.

On the other hand, low-density polyethylene (LDPE) is the second most consumed plastic, representing 17% of global plastic consumption [16]. LDPE is the cheapest thermoplastic polymer and differs from high-density polyethylene (HDPE) in its chain structure, which contains more branching. This structural difference results in lower bond strength and resistance compared to HDPE. However, LDPE has certain advantages in co-pyrolysis processes. LDPE exhibits a lower density, meaning fewer molecules/moles per unit mass. This characteristic allows for easier handling and processing of LDPE during co-pyrolysis. Moreover, co-pyrolysis of LDPE with cellulose has demonstrated a significant synergic effect, with a synergic coefficient of +83% in liquid yield [19]. It indicates that LDPE can enhance the overall liquid yield during co-pyrolysis, leading to improved oil performance and higher product yields when co-pyrolyzed with various biomasses such as cedar, sunflower stalk, and Fallopia Japonica stem [19].

As for HDPE, although it possesses a higher density and stronger intermolecular forces than LDPE, it still has its advantages in co-pyrolysis applications. HDPE is known for its high strength–density ratio, making it a durable and robust thermoplastic. These characteristics contribute to its suitability for diverse industrial applications. HDPE is the fourth most-produced plastic worldwide in volume, indicating its widespread availability and accessibility for co-pyrolysis processes [16]. However, further investigation is needed to determine the specific advantages of HDPE in co-pyrolysis and its potential synergistic effects with different biomass feedstocks [20]. The primary objective of this study was to assess the impact of operational parameters on gas composition during non-catalytic co-hydropyrolysis. The focus was on investigating the effects of varying parameters on mixtures of Chilean Oak sawdust, HDPE, and LDPE, aiming to minimize oxygenated compounds in the gas stream. The study systematically varied the temperature, heating rate, ChO/plastic ratio, and hydrogen pressure to understand their individual and combined effects. GC/MS analysis identified variations in oxygenated compound levels.

Furthermore, the study of biomass and plastic co-hydropyrolysis processes is relatively new and has shown promising results in waste valorization. The novelty of this study lies in the gas phase analysis and the determination of the main operational factors that have a significant impact through a robust experimental design. This research provides a fresh approach to understanding how these factors interact, affecting the formation of oxygenated compounds. Additionally, a comprehensive reaction mechanism for co-hydropyrolysis is proposed by analyzing the complex interactions and chemical reactions between the biomass and plastic components.

This mechanistic understanding provides valuable insights into the fundamental aspects of the co-hydropyrolysis process. The findings of this study contribute to understanding co-hydropyrolysis and offer insights for minimizing oxygenated compound formation. This knowledge contributes to optimizing process conditions and designing more efficient biomass and plastic valorization systems in future studies.

## 2. Materials and Methods

### 2.1. Raw Material

Chilean native Oak (ChO, *Nothofagus obligua*) was provided by Miraflores Angol Ltd. in Angol, Chile. The samples underwent a size comminution process by sawing, chipping, grinding, and sieving to reach a particle diameter range of 0.125 ≤ d_p_ ≤ 0.225 mm. The high- and low-density polyethylene (HDPE and LDPE) used were virgin polymer beads donated by UDT, Concepcion, Chile. They were subjected to a size reduction process by melting, grating, and sieving for a granulometry between 0.125 and 0.225 mm.

### 2.2. Experimental Setup

ChO/plastic co-hydropyrolysis was conducted in a Py-GC/MS system consisting of CDS Pyroprobe 5200HPR (CDS Analytical, Oxford, PA, USA) coupled to a Perkin Elmer Clarus 690 chromatograph (Cambridge, MA, USA), connected with a Perkin Elmer Clarus SQ-8T MS Detector (Cambridge, MA, USA). About 0.5 mg of the samples (considering ChO/plastics ratios reported in Table 1) was prepared and introduced into a quartz pyrolysis tube filled with quartz wool on both sides. Then, the quartz tube was placed into a Pt coil to be pyrolyzed at 550 °C in a controlled atmosphere (H_2_), temperature, heating rates, and isothermal pyrolysis time (15 s). The evolved pyrolysis compounds were initially trapped in a Tenax Trap, which was then heated up to 280 °C to send it to the GC/MS system through a transfer line (280 °C). Next, an Elite 1701 column (30 m × 0.25 mm × 0.25 μm) heated from 45 to 280 °C, with He as the carrier gas at 15 mL·min^−1^, allowed for compound separation and their final identification by comparison of their mass spectra with an m/z range of 35–300 Da and compared with the 2017 NIST MS library. 

### 2.3. Thermogravimetric Analysis 

Thermogravimetric experiments were conducted in a NETZSCH thermobalance ST409PC (Pomerode/SC, Brazil) by heating (10 °C·min^−1^) 10 mg of the samples (considering the ChO/plastics ratios reported in Table 1) from 100 to 700 °C with a hydrogen flow of 20 mL/min. The sample mass was registered continuously as a function of temperature and time. The synergistic effect on the mixtures was evaluated using the Δ*W* factor, which compares the experimental and theoretical TG curves. The calculated profile was determined from a linear relationship between the experimental TG values of Oak, HDPE, and LDPE, according to Equation (1).

(1) $$\Delta W=W_{M}-\alpha \times W_{Biomass}+\left(1-\alpha \right)\times W_{Plastic}$$

where Δ*W* is the synergistic effect, *α* is the mass fraction, and *W_M_* is the experimental residual mass of each mixture.

### 2.4. Experimental Design

The analyzed independent variables and their coded levels for the experimental design are described in Table 1. 

First, the final pyrolysis temperature was set at 550 °C for 15 s. Then, a comprehensive series of experiments were performed, encompassing various configurations that included ChO, plastics, and different ChO/plastics ratios (2:1, 1:1, and 1:2). To investigate the impact of different heating rates, slow-pyrolysis (12.5 °C/s) and fast-pyrolysis (10 °C/ms) conditions were employed. The hydrogen injection pressure inside the pyroprobe reactor was 100, 150, and 200 psi. Fifty-four experiments were carried out: 36 using biomass/plastic mixtures and 18 with pure substrates under the same H_2_ pressure and heating rate conditions. Minitab software randomly planned such experiments [21]. The gas-phase chemical composition of each experimental run was analyzed using TurboMass Software 6.1. Initially, chemical compounds were identified using the National Institute of Standard and Technology (NIST) library and categorized into distinct families. The significant groups identified included acids, alcohols, aldehydes, hydrocarbons, ketones, and phenols. Subsequently, a compositional analysis was conducted for each family and individual compound using the coded levels specified in Table 1.

### 2.5. Synergy Coefficient Analysis

Synergy in co-pyrolysis refers to the phenomenon where the interaction between different feedstocks during pyrolysis produces a notable effect that goes beyond the sum of their individual contributions. In ChO/polyethylene co-pyrolysis, synergy is determined by comparing experimental values with theoretical calculations using Equations (2) and (3). These equations provide a means to quantitatively assess the contribution of each feedstock in the overall process. The synergy effect (Δ*Y*) is considered positive when the percentage of oxygenated compounds observed during co-pyrolysis is lower than expected based on the individual contributions of ChO and polyethylene. It indicates that the interaction between ChO and polyethylene reduces oxygenated compounds’ formation compared to their independent pyrolysis. A positive synergy effect suggests a beneficial interaction between the feedstocks, resulting in improved pyrolysis performance and potentially enhanced product yields.

(2) $$Y_{t}=\alpha \times Y_{Biomass}+\left(1-\alpha \right)\times Y_{Plastic}$$

(3) $$\Delta Y=\frac{Y_{t}-Y_{e}}{Y_{e}}\times 100\%\;$$

where *Y_Biomass_* and *Y_Plastic_* are the percentual content of the chemical group, *Y_t_* is the theoretical value of the mixture weighted by the weight of each fraction, and *Y_e_* is the value measured by the GC/MS system described in Section 2.2.

By quantifying and evaluating the synergy effect, it becomes possible to understand the complex interactions occurring during co-pyrolysis and assess the effectiveness of the combined feedstocks in reducing the formation of undesirable oxygenated compounds. This information is crucial for optimizing the co-pyrolysis process and designing more efficient systems for valorizing biomass and plastic waste. Considering the significant impact of the heating profile on the gas composition results in co-pyrolysis [19], an additional set of 27 experiments was conducted. These experiments involved utilizing a heating rate of 25 °C∙s^−1^ for both the mixtures and pure feedstocks while strictly maintaining the identical experimental conditions outlined in Table 1.

## 3. Results

### 3.1. Thermogravimetric Analysis

**Figure 1:**
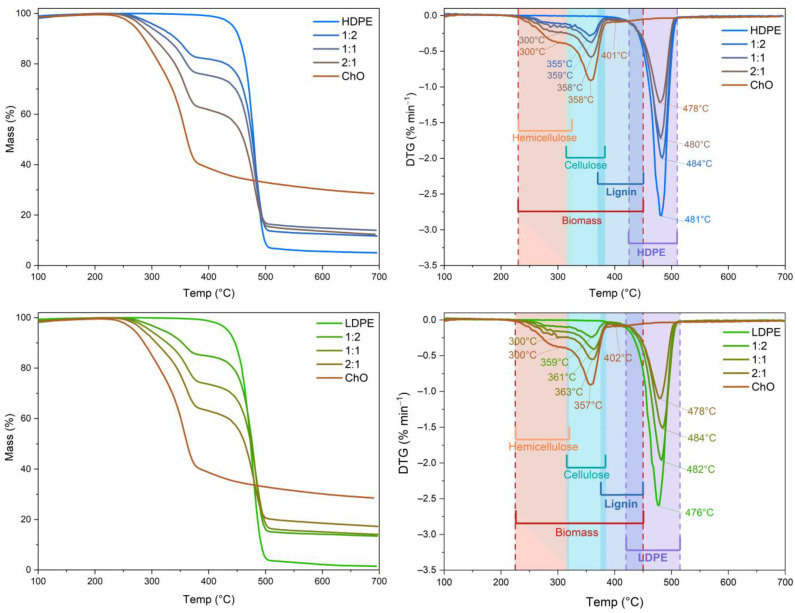
TG and DTG curves for ChO, HDPE, LDPE, and ChO:plastic mixtures.

The thermogravimetric analysis provided insights into the pyrolysis behavior of Chilean Oak (ChO), high-density polyethylene (HDPE), and low-density polyethylene (LDPE) at various mixing ratios (1:2, 1:1, and 2:1) under a hydrogen atmosphere, simulating co-hydropyrolysis. The pyrolysis of the biomass showed three distinct stages: drying, devolatilization, and decomposition of thermally stable residues [22]. Figure 1 illustrates the mass loss of Chilean Oak within the temperature range of 250–400 °C, with a peak rate of weight loss observed at 360 °C (Figure 1). The DTG curve of ChO exhibited two peaks, representing the degradation of hemicellulose and cellulose in the active pyrolysis zone, along with a tail zone associated with lignin degradation in the passive pyrolysis zone [23]. Below 250 °C, the simpler molecules decomposed (lipids and short-chain amino acids, among others), which had a negligible effect on the overall sample mass loss. Previous research by Chen et al., 2020 reported approximately 30% mass loss at temperatures below 600 °C for pure lignin [24]. In contrast, the ChO/plastics samples showed different degradation temperatures (Figure 1), primarily within the 370–500 °C range, with a maximum mass loss observed at 480 °C for both HDPE and LDPE. The significant mass loss of the plastics indicated that the pyrolysis process was completed below 500 °C [24,25]. For the ChO/plastics mixture, the thermal degradation process consisted of two stages: 250–380 °C with a peak at 365 °C and 390–500 °C with a peak at 490 °C, as depicted in Figure 1. The distinct DTG curves displayed a shoulder, a peak, and a tail, suggesting the involvement of multiple reactions during sample degradation, as reported elsewhere [26].

Zhu et al., 2006 analyzed HDPE, LDPE, and pine sawdust individually and mixed, showing very similar behavior during pyrolysis regarding mass decrease evaluated by thermogravimetric analysis. The temperature range (~390 °C) for the thermal degradation of biomass is lower than for polyethylene (~480 °C). In both cases, more than 90% of the initial mass had been lost at 500 °C. Mass loss for mixtures is significant at lower temperatures, close to 350 °C [27]. The maximum values in the DTG curve for the tested materials were HDPE > LDPE > ChO. At 500 °C, LDPE exhibited a higher mass loss than HDPE and ChO because HDPE has a more compact molecular arrangement and a greater capacity to undergo chemical reactions through free radicals.

**Figure 2:**
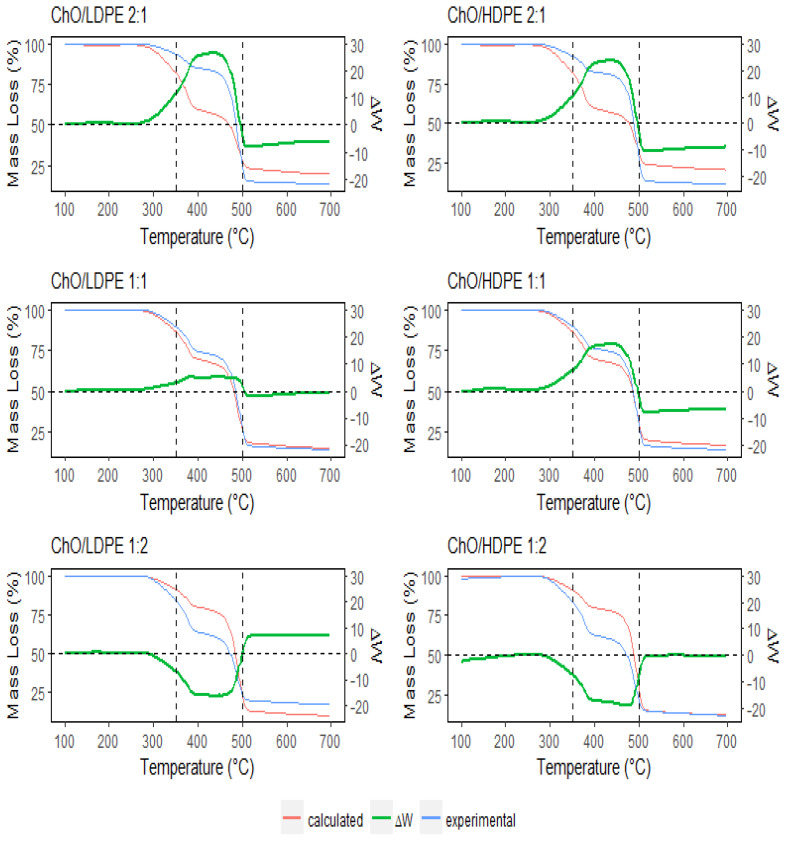
Experimental and calculated TG curves and Δ*W* for the ChO/LDPE and ChO/HDPE mixtures.

The experimental, calculated, and Δ*W* factor curves are shown in Figure 2. Initially, up to 300 °C, no significant interaction was observed, as the pyrolysis effects on ChO and the plastics were weak. However, above 350 °C, a noticeable interaction became apparent, coinciding with the temperature range of peak oak DTG (Figure 1). In the case of the 2:1 and 1:1 ChO/plastic mixtures, the Δ*W* profile exhibited an increasing trend until reaching a maximum value (between +20% and +30%). Subsequently, it decreased and became negative at temperatures above 500 °C, indicating complete pyrolysis. Positive Δ*W* values indicate that the experimental results surpassed the calculated values, suggesting a slower pyrolysis reaction. Thus, it could be attributed to heat transfer limitations, likely caused by the low conductivity of the resulting biochar being the main limitation in the reaction [28] and the constraints in the dehydration and decarboxylation reaction rates [29]. Conversely, in the 1:2 mixtures, a negative Δ*W* was observed, indicating increased reaction rates between PE radicals and ChO [24]. 

### 3.2. Experimental Design Analysis

The impact of the variables was quantified by determining the number of standard deviations of the effects, and the summarized results are presented in Table 2. The plus and minus signs indicate an increase or decrease in the percentage composition of each group or compound when transitioning from the lowest to the highest level of the coded variables. For instance, increasing biomass resulted in higher amounts of acids, aldehydes, hydrocarbons, ketones, and phenols. 

The heating rate was found to have a positive effect, increasing the proportions of acids, alcohols, ketones, and lower hydrocarbons. Conversely, hydrogen pressure emerged as a significant variable, causing an elevation in aldehyde, hydrocarbon, and ketone levels while reducing the amount of alcohols and phenols. Moreover, the type of plastic employed exhibited significance only concerning the alcohol content, resulting in a reduction when ChO was mixed with HDPE. Additionally, the interaction between factors B and C, as well as C and C, demonstrated a positive effect, which was particularly notable in the case of the alcohols. Conversely, the C and C interaction had a negative impact on the ketone and phenol levels.

**Figure 3:**
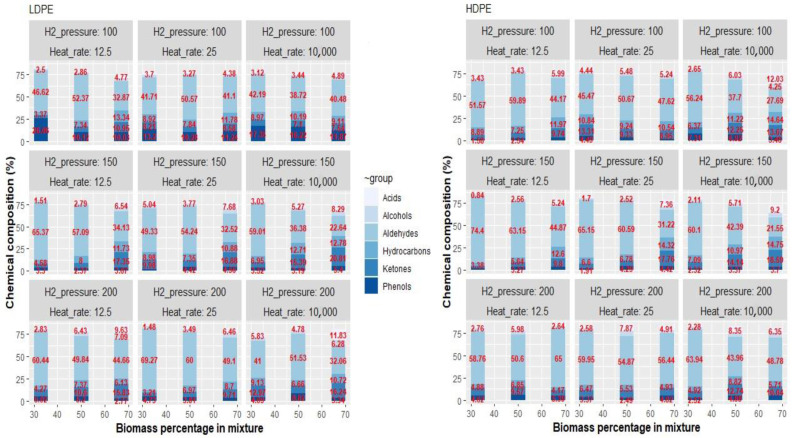
Gaseous compounds distribution.

A detailed breakdown of the compound families can be observed in Figure 3. The presence of acids tended to be more pronounced at higher heating rates and when ChO constituted 67% of the mixture. Pyrolysis of HDPE yielded a greater content of hydrocarbons compared to LDPE. However, co-pyrolysis of ChO/LDPE resulted in higher levels of alcohols, ketones, phenols, and total oxygenated compounds. On average, Chilean Oak exhibited an oxygenated compound content of 70.13%, LDPE of 5.9%, and HDPE of 1.4%. While the production of ketones and phenols from plastic materials was insignificant at a heating rate of 12.5 °C·s^−1^, increasing the heating rate to 10 °C·ms^−1^ and maintaining a hydrogen pressure of 100 psi led to values reaching 2–3%. This indicates that the hydrogen transfer reaction of the polymer was influenced under these conditions.

A comparison of these values with those obtained for Chilean Oak reveals that ketones, phenols, and acids exhibit average values of 21.4%, 20.3%, and 14.3%, respectively, whereas plastics generate less than 1%. The total content of oxygenated compounds was estimated by considering the percentages of aldehydes, ketones, and phenols. Notably, the highest values were observed for the 2:1 ChO/plastic ratio. The influence of hydrogen pressure and the heating rate is partially offset by the positive and negative impacts discussed in Table 2. However, it is essential to highlight that an increase in hydrogen pressure and heating rate generally leads to a higher concentration of hydrocarbons in the gas stream. 

Similarly, it was observed that LDPE generated fewer hydrocarbons in all mixtures, while HDPE resulted in lower aldehyde values. This trend was also evident for phenols and ketones, which tended to be higher when the plastic content in the mixture was lower and at higher heating rates, explaining the overall pattern observed. In line with these findings, Liu et al., 2019 suggested that the presence of hydroxyl radicals formed during lignin decomposition, along with small molecules produced from polyethylene degradation, contribute to the formation of phenols, furans, and aldehydes. Notably, the main components identified in the co-pyrolysis process were hydrocarbons, alcohols, and phenols, with a synergistic effect that enhanced the bio-oil yield [30]. Acetic acid and furans are cellulose and hemicellulose products formed through dehydration reactions [31]. 

However, certain compounds, such as ethylbenzene, were observed to be produced in higher quantities than expected during co-pyrolysis with HDPE. Conversely, xylene, toluene, and benzene exhibited decreased levels compared to individual pyrolysis [32]. Additionally, Oladunni et al., 2021 reported the presence of unsaturated hydrocarbons (C=C) and aromatic hydrocarbons in the pyrolysis of LDPE, along with the detection of free OH radicals, reactive carbonyl groups, aldehydes, ketones, esters, and conjugated carboxylic acids through FTIR analysis [33]. This finding is supported by a previous study by Kayacan and Dogan (2008), who observed that aliphatic (C-C and C-H) and CH2 groups were the predominant components in the pyrolysis of raw and waste HDPE and LDPE [34].

Likewise, in the ChO/LDPE and ChO/HDPE mixtures, the range of oxygenated compounds was found to be 16.1% to 52.2% and 10.7% to 51.4%, respectively. A clear trend of increasing oxygenated compounds was observed with a higher proportion of ChO in the mixture and at higher heating rates. Among the significant effects analyzed for the individual compounds (Table 2), an increase in ChO percentage, hydrogen pressure, and the heating rate resulted in higher presence of light olefins (C_2_–C_5_). Moreover, increased H2 pressure positively affected short-chain olefins (C_6_–C_10_). Previous studies on oak pyrolysis have reported that the addition of PE leads to a decrease in the total content of phenols, aldehydes, ketones, and furans while increasing the percentage of hydrocarbons, which aligns with the findings of this study. However, those studies have not thoroughly elucidated the effects of process operating conditions [35]. Additionally, incorporating HDPE in the co-pyrolysis of biomass has increased the formation of furans, acids, and methyl or propenyl phenols through dehydration and hydrodeoxygenation reactions. Consequently, the collected bio-oil exhibited a higher H/C ratio and calorific value due to a significant reduction in oxygen content [36]. 

**Figure 4:**
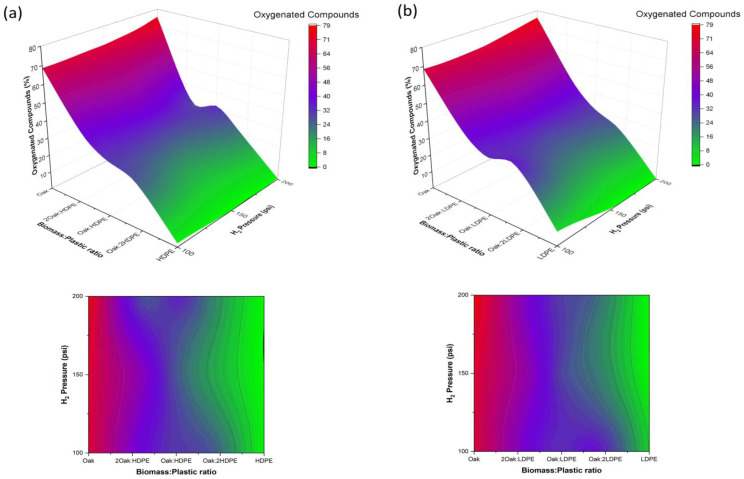
Influence of hydrogen pressure and ChO/plastic ratio on the abundance of oxygenated compounds. (**a**) ChO and HDPE mixtures; (**b**) ChO and LDPE mixtures.

Figure 4 presents the response surfaces illustrating the relationship between the ChO/plastic ratio, hydrogen pressure, and the content of oxygenated compounds. It is evident that as the proportion of plastic increases in the mixture, there is a significant reduction in the concentration of oxygenated compounds. However, an interesting finding is observed in the 1:1 ratio, where the impact of hydrogen injection into the reactor reaches its maximum synergistic effect. Thus, it generates oxygenated compounds at comparable levels obtained with the 1:2 ChO/PE mixture, with a higher plastic content of 67. This observation further confirms the findings presented in Table 2, as both the ChO/plastic ratio and hydrogen pressure were identified as highly influential factors for the formation of 2-furan-methanol, as well as the families of aldehydes, hydrocarbons, ketones, and phenols.

### 3.3. Synergistic Effect

**Figure 5:**
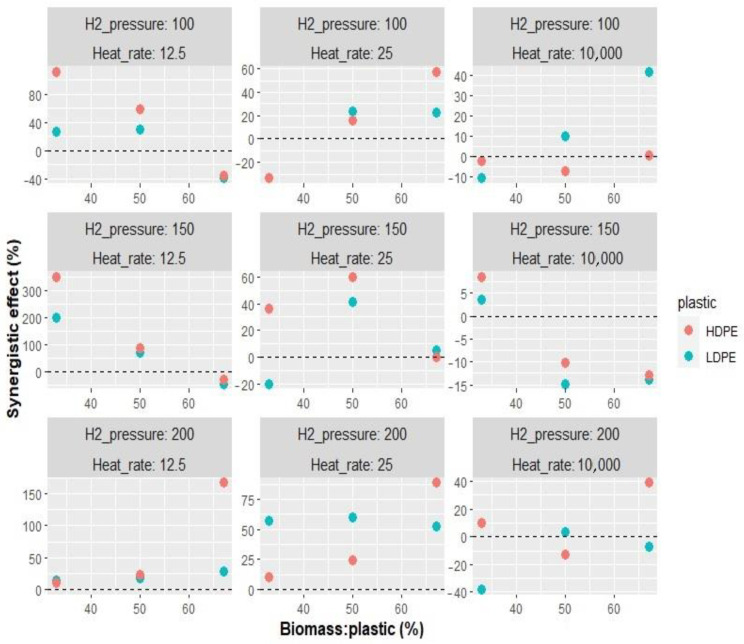
Synergistic effect in mixtures of ChO/LDPE and ChO/HDPE.

As indicated in the methodology section, a synergistic effect (Δ*Y*) with a positive value means that a more significant reduction in oxygenated compounds than expected was obtained during pyrolysis. Significantly, synergies were obtained at lower reactor heating rates at 150 psi H_2_ pressure, with values up to 350% for HDPE and 200% for LDPE. However, a synergistic coefficient of 75% was reached with HDPE and 63% with LDPE for a 25 °C·s^−1^ heating rate. Finally, at 10 °C·ms^−1^, a synergistic value of 40% was achieved with both plastics. In most experiments, a higher effect was obtained with HPDE. The average synergistic coefficient was 37.7% for HDPE and 18.0% for LDPE, as shown in Figure 5. 

Nevertheless, the observed values were higher than expected, generating a negative synergy coefficient. Specifically, two factors caused these values: a higher amount of ChO and a higher ramp rate. The lowest values are −45% with ChO/LDPE 1:2, 150 psi, and 12.5 °C·s^−1^, and 38% with ChO/HDPE 1:2, 200 psi, and 10 °C·ms^−1^. On average, at 12.5, 25, and 10 °C·ms^−1^, the reduction in oxygenated compounds was 58.3, 25.9, and −0.8%, respectively. The remarkable difference between the value expected by the additive rule of the synergistic coefficient and the experimental value of the product composition has been reported elsewhere. Hassan et al., 2019 analyzed mixtures of HDPE and sugarcane bagasse, finding differences of up to +400% in the alcohol content, +50% in the case of hydrocarbons, and −350% in the case of acids, considering that a positive value indicates that the experimental value is higher than the theoretical value [37]. Such behavior might be explained by the interaction between the HDPE-derived hydrogen and biomass hydroxyl radicals, increasing biomass decomposition during pyrolysis [38].

On the other hand, the limited effect of the synergy should be highlighted, which is limited to a specific range of biomass/plastic mix and depends on the operational conditions. Thus, Hassan et al., 2019 indicated that the hydrocarbon value was higher than expected, up to ratios of 40% HDPE. On the other hand, if the plastic percentage was higher than 40%, the hydrocarbon fraction was lower than expected. Such hydrocarbon content increase is accompanied by lower acids, improving the bio-oil quality by increasing stability and calorific value and reducing corrosion problems. An elemental analysis performed on the mixture with 40% HDPE reported +14% for carbon and −50% for oxygen and a calorific value of 42 MJ/kg (43% higher than calculated), similar to the commercial diesel reported values [33,37].

Regarding the phase distribution of the product, Hassan et al., 2020 investigated the co-pyrolysis of sugarcane bagasse with HDPE. An improvement in the liquid fraction of Δ*Y* = +6% with 60% HDPE in the mixture and Δ*Y* = +14% with a catalyst was observed [39]. Chen et al., 2016 analyzed the influence of adding HDPE in mixtures with waste newspaper. The increase in the oil phase was Δ*Y* = +32%, compared with individual experiments of HDPE and waste newspaper. In addition, the viscosity and the total acid number decreased by 76 and 216%, respectively [40]. The same trend shown in this study occurs in mixtures of Chinese pine sawdust with HDPE and LDPE, with a more significant synergistic effect in 1:1 of biomass/HDPE (+12% at 650 °C) concerning biomass mixtures with LDPE (+6% at 650 °C) [27]. 

### 3.4. Reaction Mechanism Approach

**Figure 6:**
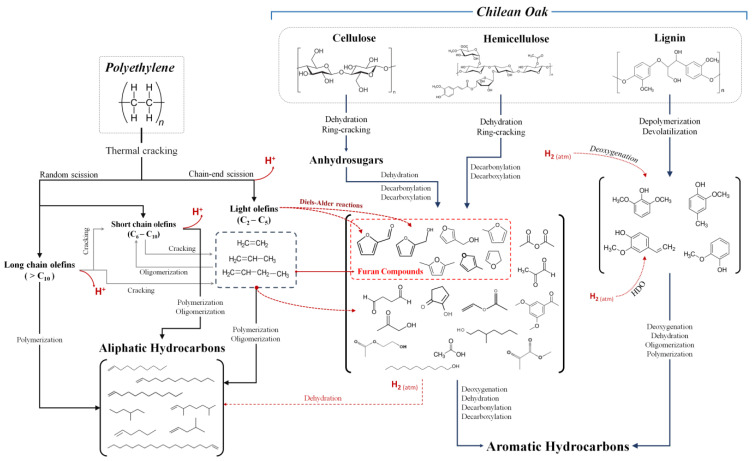
Elucidated reaction mechanism for the co-hydropyrolysis of ChO/PE.

The influence of hydrogen pressure and the ChO/plastic ratio has been described in detail in the previous sections. However, the contribution of the experimental design should be highlighted as it allowed for the analysis of these variables’ influence on the response, i.e., the composition of the gas phase produced by co-hydropyrolysis. The volatilized chemical compounds undergo a series of chemical reactions, as proposed in Figure 6. Based on the distribution of gaseous chemical compounds and previously reported studies for the formation of target species, an initial approach to the possible routes in the reaction mechanism for the non-catalytic co-hydropyrolysis of Chilean Oak and polyethylene is proposed here. As expected in lignocellulosic biomass, the thermal decomposition of Chilean Oak depends on the main fractions: cellulose, hemicellulose, and lignin. The composition of ChO was 35.38% cellulose, 35.55% hemicellulose, and 27.10% lignin, and the aqueous extractives were less than 2% [26]. Cellulose and hemicellulose are thermally degraded to anhydrosugars, aldehydes, ketones, alcohols, acids, furans and pyrans, and other oxygenated compounds by decarbonylation, decarboxylation, and dehydration reactions. Likewise, lignin degradation has been identified as the primary source of phenolic compounds [41,42,43].

Polyethene undergoes a degradation process at high temperatures in a hydrogen atmosphere through two simultaneous mechanisms: random scission and chain-end scission [44]. Since random cleavage primarily yields polyethylene-derived hydrocarbons, chain-end cleavage yields hydrogen and free radicals that are transformed into linear hydrocarbons through polymerization, oligomerization, and hydrogen transfer reactions. In addition, the hydrogen produced in the thermal degradation of the plastic can affect the oxygenated compounds derived from the biomass, which act as strong acceptors of hydrogen and promote the degradation of the PE, generating olefins and favoring the de-oxygenation of the volatilized fraction. Another reaction route suggests that the reactive hydrogen released from the plastic can react with the phenolic radicals, favoring de-oxygenation. Likewise, lignin derivatives react with hydrogen from the atmosphere through hydrodeoxygenation (HDO) reactions, which are precursors to forming aromatic hydrocarbons, as previously reported in [44,45].

The compositional analysis indicated the presence of furanic compounds (mainly furfural, 2-furan-methanol, and 2(5H)-furanone), which can react with short-chain olefins (C_2_–C_5_) released from polyethylene to form aromatic hydrocarbons through Diels–Alder reactions, followed by dehydration reactions [45]. Likewise, due to the presence of hydrogen (from the atmosphere and plastic), aromatics can be transformed through hydrogenation, decarbonylation, and decarboxylation reactions into aliphatic hydrocarbons [46]. Furanic compounds were obtained from cellulose degradation and hemicellulose depolymerization, mainly dominated by dehydration, decarbonylation, and decarboxylation reactions under 550 °C [45]. The proposed reaction mechanism indicates that the first stage during co-pyrolysis is forming radicals from the biomass, allowing the splitting of polymeric components. This must be contrasted with the results of the composition of the pyrolysis products [27,47,48]. For the second reaction stage, the hydrogen transferred from the plastic chains to the biomass-derived radicals generates cellulose decomposition and a significant mass loss [49]. HDPE has an activation energy of 517 kJ/mol and LPDE has a value of 270 kJ/mol, which is explained by the higher density of HDPE and a higher number of chains in LDPE since HDPE has a more crystalline structure [50]. Thus, the C_3_, C_4,_ and C_6_ olefins are higher in the case of LDPE [51]. Hydrogen transfer from HDPE chain cleavage can promote cellulose decomposition, and cellulose bond-breaking along with HDPE cracking is promoted by oxygenates from cellulose; this generates the synergistic effect between free radicals released by biomass and plastics. Studies on the co-pyrolysis of cellulose and HDPE in ratios of 3:1, 1:1, and 1:3 showed better results for 1:3 in volatile small molecules. Finally, the production of oxygenated compounds was suppressed while the generation of alkanes and alkenes was promoted [52]. 

## 4. Conclusions

The co-hydropyrolysis of Chilean Oak (ChO) and LDPE demonstrated a reduction in the levels of alcohols, ketones, phenols, and total oxygenated compounds compared to conventional pyrolysis. ChO exhibited an average oxygenated compound content of 70.13%, while LDPE and HDPE had significantly lower values of 5.9% and 1.4%, respectively. Experimental assays on both plastics at a heating rate of 10 °C·ms^−1^ and 100 psi of hydrogen pressure resulted in a remarkable decrease in ketones and phenols to 2–3%. During ChO hydropyrolysis, the average values of ketones, phenols, and acids were 21.4%, 20.3%, and 14.3%, respectively, while plastics generated less than 1% of these compounds. The total content of oxygenated compounds was highest for the 2:1 ChO/plastic ratio, with ChO/LDPE and ChO/HDPE mixtures exhibiting ranges of 16.1 to 52.2% and 10.7 to 51.4%, respectively. It was observed that higher proportions of biomass and heating rates increased oxygenated compound levels.

Significant synergistic effects were observed during co-hydropyrolysis, resulting in reduced levels of oxygenated compounds compared to expected values during conventional pyrolysis. For HDPE at lower heating rates and a H_2_ pressure of 150 psi, reductions of up to 350% were achieved, while LDPE demonstrated reductions of up to 200%. At a heating rate of 25 °C·s^−1^, HDPE exhibited a synergistic coefficient of 75% and LDPE exhibited a synergistic coefficient of 63%. Similarly, at 10 °C·ms^−1^, both plastics achieved a 40% reduction, with HDPE showing a higher synergistic coefficient of 37.7% compared to LDPE (18.0%).

Finally, this study contributes to the co-hydropyrolysis of biomass/plastics by demonstrating the effectiveness of the process for reducing oxygenated compounds. The results highlight the potential of LDPE and HDPE as valuable feedstocks for co-hydropyrolysis, offering opportunities for the sustainable valorization of plastic waste. The observed synergistic effects further emphasize the importance of optimizing operational parameters to enhance the efficiency of the co-hydropyrolysis process. These findings provide valuable insights and pave the way for future studies aiming to improve the co-hydropyrolysis process and reduce the production of undesired by-products.

## Figures and Tables

**Table 1:** Operational parameters, levels, and coded variables.

Operational Parameter	Levels	DoE Coded Variables
Plastic type	ChO:LDPE	−1
	ChO:HDPE	+1
ChO:plastics	1:0	
	2:1	−1
	1:1	0
	1:2	+1
	0:1	
Hydrogen pressure (psi)	100	−1
	150	0
	200	+1
Reactor heating rate (°C/s)	12.5	−1
	25.0	
	10,000	+1

**Table 2:** Summary of significant effects on individual compounds and families.

Compound Group	A	B	C	D	AC	AD	BC	BD	CC	ACC
**Acids**	++			+						
Acetic acid	++			++			−			
Propanoic acid, 2-oxo-, methyl ester	+		−−					+	−	
Acetic anhydride			++		++				++	
**Alcohols**		−−	−−				++		++	
1,2-Ethanediol, monoacetate				+	−−					
2-Furanmethanol	++		++							
**Aldehydes**	++		++							
Furfural	++		++				−			
Succindialdehyde	++		++	+				+		
**Hydrocarbons**	++		+	−						
1-Pentene, 4-methyl	++		++	++					++	
1-Tridecene				−						
1-Nonene			++			−	−			
1-Octene,3,7, dimethyl			++	−						
**Ketones**	++			+					−	
2-Propanone, 1-hydroxy-	++		++				−			
3-Methylcyclopentane-1,2-dione			++	+						
**Phenols**	++		−−						−	
Phenol, 2,6-dimethoxy-	++		−−		−					
Creosol	++			+			−			
Phenol, 2-methoxy-	++		−	++			−			
2-Methoxy-4-vinyl phenol	++		−−		++					
**Oxygenated compounds**	**++**			**+**						**−−**

++,−− Standardized effect above 3; +,− Standardized effect up 3; A: ChO percentage in the mixture, B: plastic (LDPE or HDPE), C: H_2_ pressure, and D: heating rate (slow or fast pyrolysis).

## Data Availability

Not applicable.
